# Mortality and chronic traumatic encephalopathy (CTE) among enforcers and non-enforcers in the National Hockey League (NHL)

**DOI:** 10.3389/fneur.2025.1566819

**Published:** 2025-04-01

**Authors:** Jeffrey S. Markowitz

**Affiliations:** Health Data Analytics, Princeton Junction, NJ, United States

**Keywords:** NHL enforcers and mortality, NHL enforcers and CTE, NHL enforcers and RHI, fighting and neurodegenerative disease, relationship between RHI and CTE

## Abstract

**Objective:**

Many NHL teams roster players whose primary responsibility is fighting with opposing players. Over time, these “enforcers” may experience repetitive head impacts (RHI), a risk factor for serious long-term health consequences including neurodegenerative disease. This study examined whether retired NHL enforcers and non-enforcers differ on two long-term health outcomes.

**Methods:**

In this matched cohort study conducted with retrospective, publicly available data, cohorts of former NHL enforcers and non-enforcers were compared on mortality, and CTE diagnosis. NHL players were deemed enforcers (ENFs, *n* = 239) if listed in a Wikipedia piece entitled “List of NHL enforcers.” A randomly selected sample of non-enforcers (non-ENFs, *n* = 239) were matched to ENFs on year of birth and the first NHL season played. Goalies and players with less than 30 games of NHL experience were excluded.

**Results:**

The matching procedure resulted in equivalent cohorts with respect to birth year (1969.9) and first NHL season played (1991.3). Significantly more ENFs had died (*n* = 23, 9.6% vs. *n* = 9, 3.8%; *p* = 0.01) and significantly more ENFs had been given a diagnosis of CTE (*n* = 7, 2.9% vs. *n* = 1, 0.4%; *p* < 0.05). While not statistically significant, age at death averaged 9+ years younger among ENFs (mean = 53.6) compared to non-ENFs (mean = 63). Players born in Canada were over-represented in the ENF cohort.

**Conclusion:**

This study found higher mortality and more diagnoses of CTE in a cohort of enforcers relative to matched non-enforcers. Given expanding evidence linking RHI to life-threatening long-term health impacts, the NHL must protect players and mandate rule changes that minimize or eliminate fighting.

## Introduction

Throughout its history, many National Hockey League (NHL) teams have rostered a player whose primary responsibility is fighting. These players, known as “enforcers,” protect the more skilled players on their teams, intimidate and “get even” with opponents, attempt to shift game momentum, and enhance team camaraderie. Fights in the NHL are bare-knuckled and can involve more than two players. Some fights have resulted in players being seriously injured. Some players’ seasons and even careers have ended because of injuries sustained during fights (1). About 9% of all NHL fights result in a concussion (2).

Repetitive head impacts (RHI) can affect various individuals, but athletes participating in contact and collision sports are at particularly high risk. This includes sports such as boxing, American football, soccer, rugby, wrestling, and ice hockey. In addition to athletes, other groups may experience repetitive head impacts, including military personnel exposed to explosive blasts, victims of violence and physical abuse and individuals who have experienced accidents or falls. These concussive and sub concussive exposures can result in long-term neurodegenerative issues including chronic traumatic encephalopathy, or CTE (3, 4). Due to its unique pathology (5), CTE can only be diagnosed post-mortem through autopsy. CTE is “…characterized by a pathognomonic lesion consisting of a perivascular accumulation of neuronal phosphorylated tau (p-tau) variably alongside astrocytic aggregates at the depths of the cortical sulci, and a distinctive molecular structural configuration of p-tau fibrils…” (6).

There is some scientific evidence of a causal link between repetitive head impacts and the development of chronic traumatic encephalopathy (7). In fact, a dose–response relationship, a key criterion for establishing causality, has been documented in several studies. These studies have shown a link between the length of time participating in certain sports and both the likelihood of developing CTE and the severity of the disease. This includes studies of American football players (8), rugby players (9), as well as ice hockey players (10).

Only two published studies have investigated the role that fighting plays in the long-term mortality of NHL players. Popkin et al. (11) found similar death rates between a matched cohort of players who played in the NHL between 1967 and 2022 with more than 50 career fights compared to those with fewer fights. These researchers also found an average age at death that was about ten years younger in the cohort of player-fighters compared to matched controls. Popkin et al. (11) note in their Discussion that 11 of the 21 enforcers who died had causes often linked to CTE pathology, such as neurodegenerative disorders, drug overdoses, suicides, and motor vehicle crashes.

Goldschmied et al. (12) did not find a relationship between fighting in the NHL and longevity among players participating in the 1957–1971 NHL seasons. Less frequent fighting in the NHL between 1957 and 1971 (12) may have played a role in the negative results reported.

## Objectives

Given the neurodegenerative risks associated with fighting and accompanying RHI, additional scientific studies are needed that assess the role of NHL enforcing and long-term negative health sequelae. This study will examine “enforcing” in the NHL as a risk factor for mortality and a CTE diagnosis. Additional research is especially warranted given the continued denials by the NHL commissioner, of a causal relationship between playing ice hockey and CTE (13).

## Methods

### Data

This is a retrospective, matched cohort study that examined equal numbers of enforcers (ENFs) and non-enforcers (non-ENFs). The study was based on publicly available databases (14, 15) and confirmable Internet reports.

### Independent variable

Enforcer status (yes/no) was the independent variable for this study. Although there is no official, objective list of enforcers, Wikipedia maintains a dynamic “List of NHL Enforcers” (41) that is comprised of present and past players with citations for most named players. The validity of this list is unknown.

### Cohorts

The players identified in the Wikipedia list of enforcers make up the ENF cohort (n = 239). One randomly selected non-ENF (i.e., players not identified in Wikipedia) with the same year of birth and the same first NHL season played was matched to each ENF player. This study focused on comparisons between 239 matched pairs of NHL players. In total, 478 NHL players were studied.

Goalies and players with less than 30 games of NHL experience were excluded from both cohorts. Goalies have vastly different ice hockey experiences relative to players at other positions and are rarely, if ever, considered enforcers. Players with less than 30 games of NHL experience would be dissimilar from players in the ENF cohort who tend to have longer playing careers and more games played.

### Dependent variables

The primary dependent variables in this study were mortality (dead/ alive) and a CTE diagnosis (present/absent). Mortality was included as a study outcome because RHI is a risk factor for neurodegenerative diseases, some of which can be life-threatening. In addition to CTE (16), the following neurodegenerative diseases can result in death: Amyotrophic Lateral Sclerosis (ALS) (17); Parkinson’s Disease (18); Alzheimer’s Disease (19).

The term “CTE diagnosis” (rather than just “CTE”) was used because there could be non-ENFs, both alive and deceased, who have/had CTE but were never officially diagnosed through postmortem examinations; currently the only means of definitively identifying individuals with CTE (3, 4). Vital status was extracted from the Hockey Reference (15) online database. Death data for this study was collected through February 1, 2024. Players who died following this date were considered alive, an unavoidable limitation of this study. Players with a CTE diagnosis were obtained from multiple publicly available Internet reports (20, 21). A secondary dependent variable was age at death.

### Matching variables

Each player in the ENF cohort was matched in a 1:1 ratio to a randomly selected non-ENF player on year of birth (± 2 years) and the first season played (± 2 years) in the NHL. These two matching variables were used to minimize the potential bias related to age, period and/or cohort effects (22). If more than one non-ENF player was available for that cohort, simple random sampling was used to select a matching player.

### Background variables

The background variables used to describe the two cohorts included body mass index (BMI), number of career NHL games played, total number of career goals scored and assists, country of birth, position played, and career penalty infraction minutes (PIMs). Higher PIMs have been considered a proxy for NHL fighting (11) because penalties for fighting can result in players being removed from play for five minutes or longer (instead of two minutes for most other penalties). Higher numbers of PIMs are generally indicative of more fighting on the ice.

BMI was calculated from players’ heights and weights using the standard formula (23). Most NHL players were born in Canada while almost all other players were born in the US, several countries in Europe, Russia, and a few other countries. To avoid analyzing small numbers of players, country of birth was dichotomized into Canada and elsewhere. Since goalies are excluded from the study cohorts, playing position was dichotomized into Forwards (i.e., Wingers and Centers) and Defensemen.

### Hypotheses

#### Primary

Compared to players in the non-ENF cohort, there will be significantly more deaths and players diagnosed with CTE in the ENF cohort.

#### Secondary

Compared to deceased players in the non-ENF cohort, age at death will be significantly younger in the ENF cohort.

## Analysis

Descriptive statistics (n’s, means, standard deviations, and medians) were provided for continuous background variables broken down by enforcer status accompanied by independent t-tests comparing ENFs and non-ENFs. Categorical background variables were compared by enforcer status using relative risk ratios, 95% confidence intervals (95% CI), with chi-square statistics and *p*-values. The primary study hypotheses (i.e., mortality and CTE diagnosis by enforcer status) were tested using the same statistical tests as the categorical background variables described above. Comparisons between ENFs and non-ENFs on age at death was accomplished using an independent t-test. An alpha of 0.05 (2-tailed) was used to declare statistical significance.

The statistical analysis was performed using SAS version 9.4 (40). PROC UNIVARIATE provided descriptive statistics (i.e., means, standard deviations, and medians). PROC TTEST generated t-statistics and accompanying *p*-values. PROC FREQ was used to generate 2 × 2 tables with the CHISQ option for chi-square tests and *p*-values. The RELRISK option was also incorporated into the PROC FREQ procedure to obtain relative risk statistics and their 95% CIs.

### Validity analysis

The independent variable for this study (enforcer status) was based on a Wikipedia list that has not been validated. A “gold standard” list of NHL enforcers does not exist; nor do any other comprehensive or objective lists. The Wikipedia piece does provide hundreds of citations in support of the enforcers included on their list (41). PIMs have been used in published research as a proxy for fighting (11), and as such, may also be a proxy for enforcing. A preliminary assessment of the validity of the Wikipedia list of enforcers was conducted focusing on empirical analysis of PIM values between players in the two cohorts.

## Results

### Bivariate analysis of matching variables

The matching procedure yielded ENF and non-ENF cohorts each of 239 former NHL players that were similar and not significantly different with respect to birth year and first season played in the NHL. As shown in Table 1, both cohorts had a mean year of birth of 1969.9 (*p* = 0.9944). The first season played in the NHL was about the same for the two cohorts (1991.4 and 1991.3 for ENFs and non-ENFs respectively) [*p* = 0.9973].

**Table 1:** Descriptive statistics and comparisons of cohort matching variables by enforcer status.

Cohort matching variables	*N*	Mean	SD	Median	T-statistic	*p*-value
Year of birth						
Enforcers	239	1969.9	13.1	1969	−0.01	0.9944
Non-enforcers	239	1969.9	13.1	1969		
First season played in NHL						
Enforcers	239	1991.4	13.5	1990	0	0.9973
Non-enforcers	239	1991.3	13.5	1990		

### Bivariate analysis of background variables

Table 2 contains comparisons between ENF versus non-ENF cohorts for continuous background variables based on independent t-tests. There were cohort differences on BMI, NHL career PIMs, goals scored and assists. The ENFs had higher BMIs than non-ENFs (27.3 and 26.2 respectively, *p* < 0.0001). Average NHL career PIM scores were much larger in the ENF cohort (1,104.7) compared to the non-ENF cohort with a mean of 369.4 (*p* < 0.0001). ENFs scored significantly fewer goals and assists during their NHL careers compared to non-ENFs (p’s < 0.0001). The mean number of NHL career games played by the two cohorts was 443.1 and 437 among ENFs and non-ENFs, respectively, and were not statistically different (*p* = 0.8445).

**Table 2:** Descriptive statistics and comparisons of continuous background variables—BMI, NHL career games played, PIMs, goals and assists—by enforcer status.

Continuous background variables	N	Mean	SD	Median	T-statistic	*p*-value
Body mass index (BMI)						
Enforcers	239	27.3	1.7	27.4	7.9	< 0.0001
Non-enforcers	239	26.2	1.4	26.3		
NHL games played (career)						
Enforcers	239	443.1	290.3	400.5	0.2	0.8445
Non-enforcers	239	437	380	328		
Penalty infraction minutes (career PIMs)						
Enforcers	239	1104.7	789.4	990	12.4	< 0.0001
Non-enforcers	239	369.4	462.1	180		
Goals scored (career)						
Enforcers	239	40.2	55.1	17	−4.4	< 0.0001
Non-enforcers	239	72.9	100.4	30		
Assists (career)						
Enforcers	239	67.8	89.7	33	−5.2	< 0.0001
Non-enforcers	239	129.8	160.2	62		

Two hundred of 239 ENFs (83.7%) were born in Canada (see Table 3) compared to 151 non-ENFs (63.2%) and this difference was significant (*p* < 0.0001). There was no significant difference between the two cohorts with respect to position played (*p* = 0.1859). About 70% of ENFs played Forward and 64% were defensemen (*p* = 0.1734).

**Table 3:** Comparisons of categorical background variables—born in Canada and playing position—by enforcer status.

Born in Canada								
	Born in Canada				Statistical results			
Enforcer status	Yes		No		Relative risk ratio	95% CI	Chi-square	*p*-value
	*N*	%*	*N*	%*				
Enforcers	200	83.7%	39	16.3%	1.3	1.18, 1.48	25.7	< 0.0001
Non-enforcers	151	63.2%	88	36.8%				

Playing position (Forwards vs. Defensemen)								
	Played forward							
Enforcer status	Yes		No		Relative risk ratio	95% CI	Chi-square	*p*-value
	*N*	%*	*N*	%*				
Enforcers	167	69.9%	72	30%	1.09	0.96, 1.24	1.9	0.1734
Non-enforcers	153	64%	86	36%				

* row percentages shown.

### Testing of primary study hypotheses

The main study results are displayed in Table 4. There were significantly more deceased players in the ENF cohort (*n* = 23 deaths or 9.6%) compared to non-ENFs (*n* = 9 deaths or 3.8%) [*p* = 0.0104] resulting in a relative risk of 2.6 (95% CI = 1.23, 5.41). Similar results were obtained with respect to CTE diagnosis by enforcer status. A total of seven ENFs had a diagnosis of CTE (2.9%) compared to one non-ENF (0.2%) which was significant (*p* = 0.0324). The relative risk ratio of having a CTE diagnosis among ENFs was seven times the risk found among non-ENFs (95% CI = 0.87, 58.8).

**Table 4:** Mortality and CTE diagnosis by enforcer status.

Mortality								
	Mortality				Statistical results			
Enforcer status	Dead		Alive		Relative risk ratio	95% CI	Chi-square	*p*-value
	*N*	%*	*N*	%*				
Enforcers	23	9.6%	216	90.4%	2.6	1.23, 5.41	6.6	0.0104
Non-enforcers	9	3.8%	230	96.2%				

CTE diagnosis								
	CTE diagnosis							
Enforcer status	Present		Absent		Relative risk ratio	95% CI	Chi-square	*p*-value
	*N*	%*	*N*	%*				
Enforcers	7	2.9%	232	97.1%	7	0.87, 58.8	4.6	0.0324
Non-enforcers	1	0.4%	238	99.6%				

* row percentages shown.

### Testing of secondary hypothesis

The 23 ENF players in this study who died before February 1, 2024, had a mean age at death of 53.6 years compared to 63 for the nine deceased non-ENF players. This 9+ year difference, however, was not statistically significant (*p* = 0.1768) (see Table 5).

**Table 5:** Age at death within deceased enforcers and non-enforcers.

Age at death	*N*	Mean	SD	Median	T-statistic	*p*-value
Enforcer status						
Enforcers	23	53.6	16.3	51.8	−1.38	0.1768
Non-enforcers	9	63	19.3	59.6		

### Wikipedia enforcer list validity

Selected statistical analyses were conducted to preliminarily assess the validity of the Wikipedia list of NHL enforcers (41). The first validity result was already displayed in Table 2. The ENF cohort had significantly more PIMs (mean = 1,104.7) compared to the non-ENF cohort (mean = 369.4) [*p* < 0.0001]. Next, the frequency distribution of all 478 PIM values was dichotomized. Just under three-quarters of players with PIM values above the median were in the ENF cohort (*p* < 0.0001). Moreover, within the upper 10% of the PIMs frequency distribution, 44 of 48 players (91.7%) were in the ENF cohort (*p* < 0.0001). Finally, there were no ENFs in the lower decile of the PIMs distribution.

## Discussion

Compared to non-ENFs, ENFs were significantly more likely to be deceased and significantly more likely to have a CTE diagnosis. Within deceased players, age at death was 9+ years younger among ENFs (though this difference was not statistically significant). Players born in Canada were more likely to be ENFs.

Previous research has provided clues regarding relationships between fighting in the NHL and adverse neurodegenerative outcomes (11). However, this is the first study to provide findings that are statistically significant with respect to enforcer role, and a diagnosis of CTE and death. Given the correlational nature of the findings, no causal arguments can be posited based on these results alone. Nevertheless, a growing body of scientific evidence, derived primarily from other non-ice hockey sports like American-style football and boxing has depicted deleterious neurodegenerative outcomes associated with RHI. Additional research in this area with larger samples is needed that primarily, or exclusively, focusses on ice hockey players, especially those playing at the NHL level.

Concussion rates among ice hockey players have rivaled rates reported among NFL players (24). Even sub-concussive impacts have been shown to be a risk factor for various neurodegenerative outcomes (25) and there is an abundance of such impacts in the NHL. During routine NHL play, enforcers are also subject to RHI that are unrelated to fighting. RHI in ice hockey can come from falls, knocks into playing boards, body and head-checks, hockey-stick incidents, and strikes by fast-flying pucks. Moreover, NHL players are likely to be faster, bigger, and stronger than skaters in most other ice hockey leagues and organizations.

While there are penalties for fighting that remove players from the ice for relatively brief stints of time in the NHL, these penalties are generally trivial compared to many other ice hockey organizations. The game- and multi-game ejections employed at US colleges, as well as professional and non-professional leagues and organizations around the world (26, 27) have reduced the incidence of fighting on the ice. Additionally, fighting results in ejections and fines in all other major US sports leagues including the National Football League (NFL), National Basketball Association (NBA) and Major League Baseball (MLB) (28–30). And for the first time in the storied history of Canadian ice hockey, one of the three major Canadian junior hockey leagues, the Quebec Maritimes Junior Hockey League (QMJHL), implemented much stricter penalties for the 2023–2024 season (31) that markedly reduced the incidence of fighting (32).

The Commissioner of the NHL denies a causal relationship between playing ice hockey and CTE. The epidemiologic criteria for establishing a causal relationship are quite demanding and require rigorous study (33). Because most CTE studies use specific convenience samples for histopathological evaluations, establishing a causal relationship is especially challenging. Nevertheless, the growing body of evidence is becoming very convincing. For example, based on a comprehensive and systematic evaluation of Bradford Hill criteria (33) for cause (an epidemiologic standard used for this purpose), Nowinski et al. (7) found “convincing evidence of a causal relationship between RHI and CTE, as well as an absence of evidence-based alternative explanations” (7). After reviewing the Nowinski et al. (7) publication, the Concussion Legacy Foundation sent a letter cosigned by 41 of the world’s leading CTE experts to the National Institute of Neurological Disorders and Stroke (NINDS), a division of the National Institutes of Health (NIH), requesting a review of the evidence for a causal relationship between RHI and CTE. In response to this letter, NINDS concluded that [CTE] “…is caused in part by repeated traumatic brain injuries” (34). NHL decision- and rule-makers should prioritize player safety by carefully considering the evidence of a causal link between RHI and CTE.

The difference of 9+ years between the ages at death of ENFs versus non-ENFs is almost identical to the difference reported by Popkin et al. (11) whose methodology focused on number of PIMs and fights rather than enforcer status. The difference uncovered in the present study, although not statistically significant, seemed meaningful but must be carefully considered. This is because individuals with CTE may have died from other causes. Also, there is likely to be selection bias among those who elect to be autopsied that could impact their longevity. Moreover, there is a high degree of variability with respect to age at death among CTE victims with some of them living into their seventies and eighties. Also, years of life must be considered alongside quality of life which could be impacted by cognitive deterioration and significant behavioral problems. CTE players who live longer could have a poor quality of life. According to scientific literature, individuals with CTE are at risk of suicide (35, 36). This can reduce the years of life for individuals that may have lived longer otherwise. Finally, there could be confounding variables that impact lifespan like genetic factors and general health. Those who do die young could have more RHI exposure and greater CTE severity. These and other factors may impact the speed of disease progression, and possibly age at death.

### Other findings

The results related to BMI and goals scored and assists were anticipated. ENFs tend to be bigger players than non-ENFs and their skill level on the ice, beyond fighting, is generally more limited.

One unexpected finding was the overrepresentation of ENFs who were born in Canada. In this study, 83.7% of the 239 ENFs were born in Canada compared to 63.2% of non-ENFs. Since its inception, about 65% of all NHL players have been born in Canada (37); a far lower percentage than 83.7%. *Post hoc* analyses indicated that all 23 deceased ENF players were born in Canada. Within the nine deceased non-ENF players, seven (77.8%) were Canadian born. In all, 30 of the 32 deceased players in this study (93.8%) were born in Canada. Also, all eight players with a diagnosis of CTE were born in Canada. Additional research is required to better understand the relationship between being Canadian born and enforcing and mortality among NHL players.

There has been a dramatic reduction in the number of NHL fights since the mid-1980s that has continued through the first two decades of the 21st century. Between the 2002–2003 and 2020–2021 seasons, the number of fights in the NHL declined from 0.64 to 0.18 fights per game, respectively (13). However, according to the Hockey News (42), fighting has been making a “comeback” during the 2021–2022 and 2022–2023 regular seasons averaging over 0.25 fights per game. In fact, there are still hundreds of fights occurring each season in the NHL. Changes in NHL policies and rules related to fighting must be carefully considered by the League, its players and the players’ association.

### Validity of Wikipedia enforcer list

The Wikipedia enforcer list validity results show large and statistically significant relationships between PIM values and enforcer status. These strong relationships in the expected direction provide some preliminary degree of face validity for the Wikipedia list of enforcers. Other study findings like higher BMIs and lower numbers of goals and assists are also consistent with what is known about enforcers.

### Limitations

The use of a Wikipedia article to identify the enforcer cohort for this study could be considered a limitation. As noted in this manuscript, the validity of this Wikipedia article is unknown. However, several factors and analyses described in this manuscript bolster confidence that the Wikipedia list is an acceptable measure of enforcer status. First, the article itself contains more than 225 references supporting the selection of players as enforcers. Second, the manuscript presents a detailed validity analysis based on empirical data focused on PIM (penalty infraction minutes) values. The results of these analyses provide encouraging results regarding the measure’s validity. Third, the measure has a good deal of face validity, as the enforcer cohort exhibits characteristics known to be associated with enforcers, including significantly higher BMIs relative to non-enforcers, significantly lower scoring, and, most importantly, significantly more PIMs. Furthermore, the main study results—higher death rates and more CTE diagnoses among enforcers—were predictable given their elevated RHI exposure and, hence, greater risk of negative long-term neurodegenerative health consequences. While there is no “perfect” way of identifying samples of NHL enforcers, every indication suggests that the selected players represent a valid cohort.

In this study, a total of 32 players died including 23 in the ENF cohort and nine in the non-ENF cohort. A total of eight players were diagnosed with CTE. These event numbers are too small to attempt meaningful multivariate logistic regressions, a limitation of this study. Statistical literature recommends that one independent predictor variable be entered for every ten events (e.g., deaths and CTE diagnoses), based on the lower number of events in the groups being studied (38). Without multivariate analyses, it is difficult, if not impossible, to definitively identify and consider confounding variables. Hence, there are likely to be variables, including ones studied as well as not studied in this paper, which may confound the relationship between enforcer status and mortality and CTE diagnosis. In general, this is a possibility in case control studies, including matched studies (39) and can lead to biased and misinterpreted results. Future CTE studies will only be able to address the identification and proper analyses of confounding variables by studying larger samples of players who (unfortunately) experience larger numbers of events.

The small number of events also reduced statistical power, which could bias the results toward the null. However, significant results were still obtained, indicating more deaths and CTE diagnoses among enforcers compared to non-enforcers. Additionally, among deceased cohort members, the age at death was nearly 10 years younger for enforcers. While this difference was not statistically significant, likely due to the small sample size, the magnitude and direction of the effect are notable.

A total of 12 statistical tests were conducted for this study with no correction for multiple comparisons. Hence, there is the possibility of false positive findings. Replication of these results, using a variety of study methodologies and larger samples will increase confidence that relationships between enforcing and adverse neurodegenerative health events are real.

There could be selection factors, independent of the NHL and fighting, that made adverse health events among enforcers more likely. An elevated BMI is one potential physical example. There may also be social and psychological factors that influence individuals to become enforcers. Some enforcers may be “fighters” off the ice. Cultural and experiential factors may help explain why some players become enforcers. For less skilled players, enforcing may be the only way to secure a place on an NHL roster.

Selection factors may also influence which individuals get a CTE diagnosis, given that a histopathological analysis of the brain is required to make a definitive diagnosis. One CTE research team notes in their study of American football players, for example, that “…public awareness of the possible link between repetitive head trauma and CTE may have motivated players and their families with symptoms and signs of brain injury to participate in [their] research” (36). Research is needed to determine whether more severely ill players with CTE and their families choose to be autopsied and how this and other possible selection factors impact which players participate in CTE brain research. In any case, CTE is likely to be underestimated, and there may be undiagnosed players within the non-ENF cohort. If so, this would make it more difficult to uncover the effects reported in this paper.

The validity analysis of the enforcer measure used in this study may be viewed as a limitation since it was not comprehensive and focused solely on relationships between enforcer status and PIMs. However, the most closely linked variable to enforcer status is PIMs. Simply put, more fights generally result in more PIMs. The number of NHL fights was not a suitable alternative measure for this study, as the definition of an NHL fight has changed over the years that cohort members played in the league and remains somewhat subjective. While the relationship between enforcer status and PIMs is not perfectly linear, PIMs serve as the best available measure for the validity analysis.

## Conclusion

This study found significant effects on mortality and a CTE diagnosis among a cohort of retired NHL enforcers compared to a matched cohort of non-enforcers. Like Popkin et al. (11) age at death was about ten years younger among deceased ENFs compared to non-ENFs. Goldschmied et al. (12) did not identify this finding although their study was confined to NHL players between 1957 and 1971. While fighting has declined dramatically in the NHL, there are still hundreds of fights during NHL seasons. Consequently, enforcing and the fighting associated with it continues to be a potential risk factor for RHI and neurodegenerative disease. To protect its players, it is incumbent upon the NHL to mandate rules that minimize or ban fighting altogether.

## Data Availability

The raw data supporting the conclusions of this article will be made available by the authors, without undue reservation.
